# Inner strengths as buffers against the impact of insecure attachment on depressive symptoms in working women

**DOI:** 10.3389/fpubh.2025.1668503

**Published:** 2025-12-03

**Authors:** Jia Jiao, Tinakon Wongpakaran, Rewadee Jenraumjit, Shirley Worland, Saifon Bunyachatakul, Bijing He

**Affiliations:** 1 Mental Health Program, Multidisciplinary and Interdisciplinary School, Chiang Mai University, Chiang Mai, Thailand; 2 Department of Psychiatry, Faculty of Medicine, Chiang Mai University, Chiang Mai, Thailand; 3 Department of Pharmaceutical Care, Faculty of Pharmacy, Chiang Mai University, Chiang Mai, Thailand; 4 Faculty of Health Sciences, University of New England, Armidale, NSW, Australia; 5 Department of Occupational Therapy, Faculty of Associated Medical Sciences, Chiang Mai University, Chiang Mai, Thailand; 6 Mental Health Counseling Center, Longquan Secondary Vocational School, Longquan, China

**Keywords:** mental health, depression, insecure attachment, inner strengths, working women, China

## Abstract

**Background:**

Depressive symptoms are often associated with insecure attachment. In China, working mothers are particularly vulnerable due to the intersecting pressures of work and familial responsibilities. Emerging evidence suggests that inner strength—including resilience, emotional regulation, and positive coping—may buffer the impact of insecure attachment on depressive symptoms in this population.

**Objective:**

The primary objective was to determine whether inner strengths moderate the relationship between insecure attachment and depressive symptoms among Chinese working mothers. A secondary objective was to assess the prevalence of depressive symptoms in this population, measured with the OI-Depression subscale (Outcome Inventory-21).

**Materials and methods:**

A cross-sectional survey was conducted with 330 Chinese working mothers aged 30–45 years. Participants completed validated measures: the Outcome Inventory for Depression, Experiences in Close Relationships, and the Inner Strength-Based Inventory. Moderation analysis was performed using the PROCESS macro, Model 1, to investigate whether inner strengths modified the relationship between insecure attachment and depressive symptoms.

**Results:**

Insecure attachment (*B* = 0.233, *p* < 0.001) and inner strengths (*B* = −0.295, *p* < 0.001) were both significantly associated with depressive symptoms. Notably, a significant moderation effect was observed (interaction *B* = −0.009, *p* < 0.001): higher levels of inner strengths were associated with a weaker positive relationship between insecure attachment and depressive symptoms. This study also found the prevalence was 38.2% for depressive symptoms in this group based on the screening tools.

**Conclusion:**

Inner strengths significantly moderated the link between insecure attachment and depressive symptoms among Chinese working mothers. These findings highlight the protective potential of enhancing inner strengths in mental health interventions for this population. Further longitudinal research is recommended to confirm these effects and explore strategies to foster inner resilience.

## 1 Introduction

The robust economic growth and rapid urbanization over the past decade have enabled a significant proportion of women to enter the workforce in China. Women have been achieving higher educational levels and securing employment opportunities as part of the ongoing economic reforms (1), which have also emphasized women's familial obligations. In Chinese society, profoundly shaped by Confucian cultural values, women have traditionally been expected to assume family-oriented roles (2). Working mothers denote females with offspring who are employed in full-time or part-time positions while concurrently shouldering primary caregiving and household responsibilities (3). However, due to the rising costs of urban livelihood, notably in housing, education, and healthcare, a growing number of households depend on dual parental incomes to maintain their living standards (4). A 2020 Chinese survey found that approximately 35% of mothers were in full-time employment, with an additional 8.4% holding part-time jobs or running side businesses (5). Particularly during the primary schooling years of children, mothers are frequently expected to offer intensive academic and emotional support (6). Work-family conflict is especially acute for midlife women (7). The mental wellbeing of working mothers constitutes a pressing public mental health issue, particularly in rapidly developing societies like China.

A finding from the Global Burden of Diseases 2019 Study indicates that the global count of individuals grappling with depression surged from 172 million in 1990 to 258 million in 2017, signifying a 49.86% growth (8). The WHO reports that around 54 million Chinese citizens are afflicted with depression (9). Depressive symptoms are highly prevalent among working mothers. A prospective investigation in Malaysia documented that mothers resuming work post-pregnancy face elevated risks of physical and psychological health impairments. The study reported a notable incidence of postnatal depression within the first 6 weeks postpartum (10) while demonstrating significant disparities in depression prevalence between working and non-working mothers, with the former group displaying a heightened vulnerability to depressive symptoms (11). Many factors are associated with depressive symptoms among women, especially among individuals who are working (12). Women exhibit a significantly higher predisposition to depression during perimenopause and the menopausal transition (13). Notably, individuals working over 55 h weekly face 1.65- and 1.68-fold increased risks of depressive symptoms during these phases (14). Moreover, chronic pain was three times more likely to meet the diagnosis of depression (15), while mothers of multiple children encounter higher parenting pressures compared to their one-child counterparts (16). Insecure attachment is also an established risk factor for depression (17).

Attachment theory, initially proposed by John Bowlby, posits that early emotional bonds with caregivers shape individuals' internal working models of self, others, and relationships, which endure throughout the lifespan (18). Insecure attachment, including anxious and avoidant attachment, has been repeatedly associated with elevated depressive symptoms. Individuals with anxious attachment typically exhibit incessant cravings for reassurance and harbor profound fears of abandonment, thereby fostering chronic emotional dysregulation that may precipitate depressive episodes. Conversely, those with avoidant attachment tend to suppress emotional needs and interpersonal struggles, which may culminate in feelings of social isolation and depressive symptoms due to their inability to foster meaningful emotional bonds (17). One study has shown that insecure attachment among working mothers can manifest in different ways. Some working mothers may experience difficulties in balancing their work and family roles, leading to feelings of guilt, anxiety, and a sense of inadequacy in their parenting, which are all signs related to insecure attachment (19). These feelings can further affect their interactions with their children, potentially leading to a less secure attachment relationship between mother and child. The relationship between insecure attachment and depression has been proven. A study investigated a large sample of adults and found that those with insecure attachment styles reported significantly higher levels of depressive symptoms compared to those with secure attachment. The study suggested that the fear of abandonment and difficulty in forming healthy connections characteristic of insecure attachment may contribute to feelings of isolation, low self-esteem, and hopelessness, all of which are core features of depression (20). Another study focused on the neural mechanisms underlying the relationship between insecure attachment and depression. Using neuroimaging techniques, they found that individuals with insecure attachment showed abnormal activation in brain regions associated with emotion regulation and self-perception, which are also implicated in the pathophysiology of depression (21).

While risk factors for women have been extensively investigated, protective factors, especially modifiable psychological factors, are limited. This research will demonstrate that inner strengths are seen as a core positive psychological change and adaptation (22), and could be one of them. In a previous study, patients with a high level of inner strength are more capable of mobilizing external resources to manage disease progression and treatment, thereby enhancing adherence to therapy and maintaining a better quality of life despite diseases (23). Another study examining inner strength identified ten positive behavioral traits, including truthfulness, perseverance, patience and endurance, wisdom, equanimity, generosity, determination, morality, mindfulness or meditation, and loving-kindness, which found that among Chinese salespersons, inner strength would have a negative correlation with anxiety (24). Even brief sessions of loving-kindness meditation can modulate brain wave activity associated with emotional regulation, particularly in beta and gamma frequencies within the amygdala and hippocampus, which may contribute to reductions in depressive symptoms (25).

**Figure 1:**
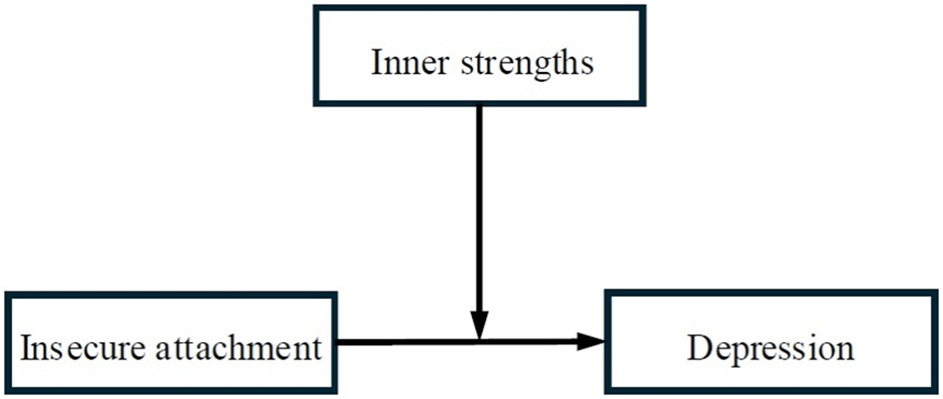
The model of inner strengths on insecure attachment and depressive symptoms.

Prior research has confirmed that both subtypes of insecure attachment predict depression in general adult samples (26), but working women's unique stressors—such as work-family conflict or gender discrimination—may intensify these links, making insecure attachment a particularly salient risk factor for this group. From a strength-based perspective, these resources enable individuals to reframe stressors, regulate negative emotions, and access adaptive coping strategies—capacities that directly counteract the maladaptive processes associated with insecure attachment. While prior research has linked inner strengths to lower depression and insecure attachment to higher depression (27), few studies have explicitly tested inner strengths as a buffer: that is, whether they weaken the association between insecure attachment and depressive symptoms in working women. This gap is notable, as working women both face heightened stressors that activate attachment-related vulnerabilities and often develop inner strengths through navigating these challenges—making them an ideal population to study this buffering effect and to test the hypothetical moderate model of inner strength (Figure 1), hoping to provide a reference for targeted interventions to improve the public mental health of vulnerable populations.

## 2 Materials and methods

### 2.1 Study design

This research adopted an online cross-sectional survey through WeChat conducted in mainland China. We used WeChat because it has over 1.2 billion monthly active users in China, covering 98% of internet users aged 18–60. Additionally, WeChat's compatibility with mobile devices (over 90% of users access it via smartphones) reduced response barriers, enabling participants to complete the survey anytime, anywhere, and leading to a higher valid response rate. Ethical approval for the research was obtained from the Research Ethics Committee of the Faculty of Medicine at Chiang Mai University. Prior to data collection, the researcher secured authorization to utilize the validated Chinese versions of all measurement tools. Following this, a full questionnaire was made, incorporating both self-administered items and all the verified instruments. A preliminary pilot study involving 30 participants was then conducted to evaluate the psychometric characteristics of the measures in the Chinese population and to calculate the average completion time. Subsequently, the administrators of the relevant WeChat groups granted the researcher permission to distribute the questionnaires via their respective platforms. Before taking part in the study, all participants provided written informed consent. The sample size (*n*) determination was based on a statistical formula informed by prior research:


$$\begin{array}{l} n=\frac{p\left(1-p\right)z^{2}}{e^{2}} \end{array}$$ (1)

With: *z* = 1.96 for a 95% confidence level (α), *p* = 0.3 for the proportion (expressed as a decimal), *e* = 0.05 for the margin of error, and *n* = 1.96^2^ × 0.3 × (1–0.3)/0.05^2^. The calculated sample size was approximately 323. To ensure robust statistical power, a total of 330 questionnaires were ultimately collected based on inclusion criteria, exceeding the minimum required sample size.

### 2.2 Participants

The participants comprised working mothers from urban areas across China. To be eligible, participants had to satisfy the following inclusion criteria (1) women aged 30–45 years; (2) mothers of one or more primary school students (aged 6–11 years); (3) individuals employed in full-time or part-time job; (4) women in pregnancy, premenopausal or perimenopausal stages; and (5) owners of computers or smartphones with reliable internet access and have WeChat. Exclusion criteria were (1) employed individuals on extended leave; and (2) residents of rural areas as per China's household registration system.

### 2.3 Data collection

A self-administered questionnaire was created using the Questionnaire Star platform and distributed in WeChat parenting groups for primary school parents, after obtaining approval from group administrators. Participants received study details, a participant information sheet, and an informed consent form. Eligibility was self-assessed via inclusion/exclusion criteria in the online survey. No private data was collected; only network identifiers were visible, and all responses were anonymized. Respondents received a CNY 5 incentive via Alipay. Some participants further shared the survey for convenience sampling. Data access was password-protected and restricted to authorized researchers, with regular audits and encrypted backups ensuring security. The survey closed upon reaching the sample target, and data will be stored securely and de-identified for about 1 year after cleaning.

### 2.4 Procedure

Each participant was provided with a comprehensive research briefing, including a Participant Information Sheet (PIS) and an Informed Consent Form (ICF). The questionnaire, developed only for research purposes, required participants to complete self-assessment items to verify eligibility based on predefined inclusion and exclusion criteria. All data were anonymized to protect participant confidentiality. In total, 369 individuals were recruited via an online platform, and 330 completed questionnaires met the eligibility criteria. Each respondent received a CNY 5 (USD 0.7) Alipay Red Envelope as a token of appreciation, with payment processed confidentially by the researcher. The first author performed daily data downloads to a secure offline database accessible to the research team. Data collection was carried out between October 17 and November 21, 2024.

### 2.5 Measurements

#### 2.5.1 Self-administered measure

Sociodemographic and socioeconomic characteristics were measured via self-administered items assessing participants' age, marital status, weekly working hours, educational level, and annual income.

#### 2.5.2 Outcome Inventory 21 (OI-21)

The Outcome Inventory-21 (OI-21) is a 21-point Likert scale measure (28) that evaluates four core domains: anxiety, depression, interpersonal difficulties, and somatization, and responses are evaluated using a 5-point scale (0 means never, 4 means always), yielding sum scores spanning from 0 to 84. Higher scores signify more symptom severity. In this research, only the depression subscale was used, with Cronbach's alpha coefficients of 0.93. The prevalence threshold was determined based on prior research, with a cut-off score of 7 denoting clinically significant depressive symptoms (28).

#### 2.5.3 Experiences in close relationships-revised (ECR-R-10)

Adult attachment is commonly evaluated via ECR-R-36 (29). For the present study, the shortened form experiences in close relationships-revised (ECR-R-10) was employed (30). This shortened instrument consists of two subscales, attachment anxiety and attachment avoidance. A 7-point Likert scale is employed to rate the responses, and scores greater than 4 (the median value) are categorized as “high” for each dimension. The ECR-R has been previously validated among Chinese populations, demonstrating strong psychometric properties in this cultural context. The Chinese adaptation of the ECR-R-10 has demonstrated reliability and validity (31) with Cronbach's alpha coefficients of 0.93 (anxiety) and 0.90 (avoidance) in this population.

#### 2.5.4 Inner-Strength-Based Inventory (I-SBI)

Inner-Strength-Based Inventory (I-SBI) (22) evaluates ten positive behavioral traits rooted in the Buddhist doctrine's 10 perfections: truthfulness, perseverance, patience and endurance, wisdom, equanimity, generosity, determination, morality, mindfulness or meditation, and loving-kindness. The scale, which consists of 10 items, adopts a 5-point Likert scale (1 represents the lowest, and 5 represents the highest). Total scores vary from 10 to 50, and higher values are indicative of greater inner strengths. The Chinese adaptation of the I-SBI (24) has exhibited excellent internal consistency, yielding a Cronbach's α of 0.86 in prior research. In the present study, the scale demonstrated comparable reliability (Cronbach's α = 0.88).

### 2.6 Statistical analysis

The IBM Statistical Package for the Social Sciences (SPSS) Version 27 was utilized to conduct data analysis. Descriptive statistical analyses were conducted to characterize sociodemographic and socioeconomic characteristics, reported as frequencies and percentages. Pearson's bivariate correlations assessed associations between study variables, followed by Multiple linear regression to examine predictors of depressive symptoms. A significance level of *p* less than 0.05 was employed to identify statistical significance, where *p* less than 0.01 and *p* less than 0.001 represented increasingly high degrees of significance.

To test whether inner strengths moderate the association between insecure attachment and depressive symptoms, a moderation model, PROCESS macro, Model 1, was employed. This analysis employed 5,000 bootstrap resamples to generate bias-corrected confidence intervals, a technique that mitigates the limitations of small sample sizes and enhances the reliability of parameter estimates, particularly when addressing non-normal distributions or limited sample representativeness.

## 3 Results

### 3.1 Sociodemographic and socioeconomic characteristics

Table 1 presents a summary of the sociodemographic and socioeconomic characteristics of the 330 Chinese mothers, along with comparisons and corresponding percentages. The sample characteristics reveal that 83.6% of participants were in marital or cohabiting relationships, while 42.7% (*n* = 141) reported having two or more children. Educational attainment was relatively high, with over half of the respondents holding at least a bachelor's degree. Among the employed mothers (*n* = 205), the majority were engaged in full-time jobs, typically working 40 h or more weekly. Regarding household economic status, around 75% of the respondents reported an annual income below CNY 150,000, suggesting that the sample predominantly consisted of a lower- to middle-income population.

**Table 1:** Sociodemographic and socioeconomic characteristics (*n* = 330).

**Variables**	**Categories**	***n***	**%**
Age	30–35	94	28.5
	36–40	141	42.7
	41–45	95	28.8
Marital status	Married/cohabiting	276	83.6
	Single	9	2.7
	Divorced/widowed/separated	45	13.6
Children number	1	189	57.3
	2	111	33.6
	≥3	30	9.1
Educational level	High school and below	81	24.6
	High vocational school	69	20.9
	Bachelor's degree and above	180	54.5
Weekly working hours	1–20	48	14.6
	21–39	77	23.3
	40–54	147	44.5
	≥55	58	17.6
Annual income (CNY)	0–60,000	78	23.6
	61,000–10,000	88	26.7
	101,000–150,000	81	24.5
	>151,001	83	25.2

CNY, Chinese yuan (7.30 CNY = 1 U.S. Dollar).

### 3.2 Pearson's correlation among variables

Table 2 presents the correlation coefficients among four key variables, involving attachment anxiety, attachment avoidance, depression, and inner strengths. Results show significant positive correlations between attachment anxiety and depression (*r* = 0.625, *p* < 0.01), as well as attachment avoidance and depression (*r* = 0.445, *p* < 0.01). Conversely, significant negative correlations exist between inner strengths and attachment anxiety (*r* = −0.418, *p* < 0.01), inner strengths and attachment avoidance (*r* = −0.393, *p* < 0.01), and inner strengths and depression (*r* = −0.708, *p* < 0.01), indicating complex associations between attachment dimensions, depression, and inner strengths related constructs.

**Table 2:** Correlation coefficients among variables (*n* = 330).

**Items**	**1**	**2**	**3**	**4**
1. Attachment anxiety	–			
2. Attachment avoidance	0.617^**^	–		
3. Depression	0.625^**^	0.445^**^	–	
4. Inner strengths	−0.418^**^	−0.393^**^	−0.708^**^	–

^**^p < 0.01.

### 3.3 Factors associated with depressive symptoms among working mothers

To explore the correlates of depressive symptoms among working mothers, a multiple linear regression analysis was used. First, all the control variables were entered, including age, marital status, and attachment avoidance. Secondly, primary predictor variables—inner strengths and attachment anxiety—were added to assess effects while controlling for covariates. Findings from the analysis (see Table 3) identified that attachment anxiety (β = 0.212, *p* < 0.001) and number of children (β = 0.547, *p* = 0.041) exhibited significant positive associations with depressive symptoms. Conversely, inner strengths (β = −0.277, *p* < 0.001) and educational level (β = −2.264, *p* < 0.001) emerged as strong negative predictors. This research found that the prevalence was 38.1% for depressive symptoms of Chinese working mothers based on a cut-off score of 7 for depressive symptoms.

**Table 3:** Factors associated with depressive symptoms among working mothers (*n* = 330).

**Variables**	***B***	**SE**	**β**	***t***	***p*-value**
Inner strengths	−0.277	0.023	−0.465	−11.852	< 0.001
Attachment anxiety	0.212	0.025	0.369	8.585	< 0.001
Attachment avoidance	−0.018	0.028	−0.027	−0.652	0.515
Number of children	0.547	0.267	0.076	2.049	0.041
Age (0 = 30–40 years old, 1 = 41–45 years old)	−0.102	0.366	−0.009	−0.279	0.781
Marital status (0 = married/cohabitating, 1 = non-married^*^)	0.645	0.998	0.021	0.646	0.519
Educational level (0 = below bachelor, 1 = bachelor and above)	−2.264	0.438	−0.199	−5.164	< 0.001
Annual income (0 = ≤ 100,000, 1 = >100,000)	0.396	0.357	0.040	1.109	0.268
Weekly working hours	0.206	0.158	0.044	1.301	0.194

^*^Single/divorced/widow/separated.

### 3.4 Tests of moderation

Table 4 examines the moderating effect of inner strengths on the link between insecure attachment (including both attachment anxiety and attachment avoidance) and depressive symptoms among working mothers. Insecure attachment (*B* = 0.233, *p* < 0.001) and inner strengths (*B* = −0.295, *p* < 0.001) had significant direct impacts on depressive symptoms. Notably, their interaction (*B* = −0.009, *p* < 0.001) showed that inner strengths weakened the positive association of insecure attachment with depressive symptoms. The model explained 65.3% of depression variance (*R*^2^ = 0.653, *p* < 0.001), indicating inner strengths buffer insecure attachment-related depressive risks.

**Table 4:** The moderation effect test of inner strengths in insecure attachment to depressive symptoms.

**Variables**	***B***	**SE**	***t***	***p*-value**	**LLCI**	**ULCI**
Constant	5.175	0.172	30.132	< 0.001	4.837	5.513
Insecure attachment	0.233	0.021	11.251	< 0.001	0.192	0.274
Inner strengths	−0.295	0.022	−13.198	< 0.001	−0.339	−0.251
Insecure attachment × inner strengths	−0.009	0.002	4.338	< 0.001	−0.014	−0.005
*R* ^2^	0.653 (*F* = 204.187, df1 = 3, df2 = 326, *p* < 0.001)					

**Figure 2:**
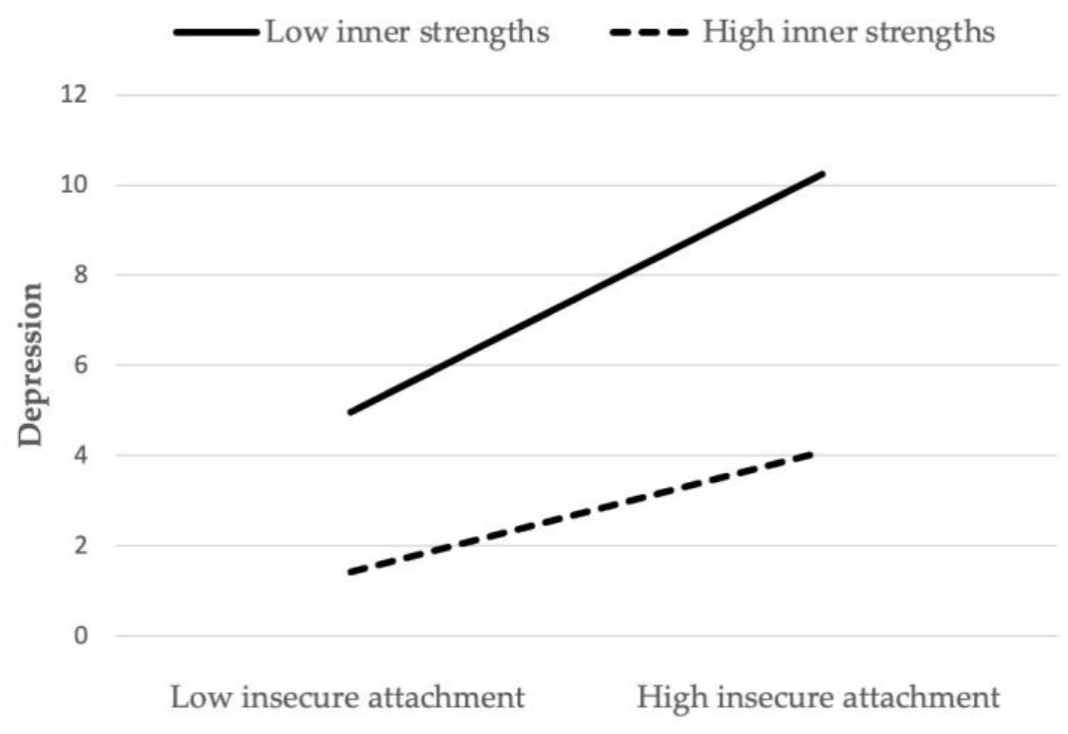
The moderation effect of inner strengths on insecure attachment and depressive symptoms.

Figure 2 presents the moderating role of inner strengths in the relationship between insecure attachment and depressive symptoms. The moderation plot quantifies these relationships: as insecure attachment increases, depressive symptoms rise more sharply with low inner strengths (solid line) compared to those with high inner strengths (dashed line). The visualization validates that inner strengths function as a protective factor, reducing the positive link between insecure attachment and depressive symptoms, and provides a cohesive depiction of how this psychological resource buffers against depressive risks in the context of insecure relational patterns.

Table 5 presents the results of the moderation effect test across different groups of inner strengths. Three subgroups were examined: M – SD, M, and M + SD. Results showed significant moderation effects in all groups: for M – SD, *B* = 0.310, *p* < 0.001; for M, *B* = 0.233, *p* < 0.001; and for M + SD, *B* = 0.156, *p* < 0.001. These findings indicate that the moderating role of inner strengths is present across different levels of the strength continuum, with the effect magnitude decreasing as inner strengths increase from M – SD to M + SD.

**Table 5:** The moderation effect of insecure attachment on depressive symptoms at different levels of inner strengths (*n* = 330).

**Level of inner strengths**	***B***	**SE**	***t***	***p*-value**	**LLCI**	**ULCI**
Low (Mean – 1 SD)	0.310	0.028	11.151	< 0.001	0.255	0.365
Average (Mean)	0.233	0.021	11.251	< 0.001	0.192	0.274
High (Mean + 1 SD)	0.156	0.027	5.817	< 0.001	0.103	0.208

SD, standard deviation; LLCI, lower limit of confidence interval; ULCI, upper limit of confidence interval.

## 4 Discussion

This study investigated how an individual's capacity to sustain emotional stability, psychological resilience, and a positive mindset has been linked to alleviating depressive symptoms. In line with Sripunya et al.'s research, the inner strength functioned as a protective mechanism against negative mental health outcomes among individuals experiencing feelings of emptiness (32). Similarly, inner strength served as a protective factor for major depression, anxiety, and somatic symptoms among Chinese sales workers (24), as well as among patients with Spinocerebellar ataxia (33). The potency of inner strength as a positive factor may stem from its incorporation of multiple virtues, including truthfulness, perseverance, patience and endurance, wisdom, equanimity, generosity, determination, morality, mindfulness or meditation, and loving-kindness. Although these virtues have a Buddhist origin, they represent universal positive traits that can be nurtured in non-Buddhist contexts. Notably, while the Inner-Strength-Based Inventory (I-SBI) was initially developed in a Thai Buddhist context, its core values are also embedded within Mahāyāna Buddhism and broader Chinese cultural and philosophical traditions. In this study, the I-SBI demonstrated excellent internal consistency. However, we acknowledge that comprehensive psychometric validation of the I-SBI has not yet been conducted in Chinese populations, and have noted this limitation in our manuscript. Further research is recommended to assess its validity in the Chinese context.

The moderating effect likely operates through two key mechanisms. First, inner strengths enhance an individual's ability to regulate emotions, reducing the emotional dysregulation triggered by insecure attachment. For example, individuals with high inner strengths may employ mindfulness or equanimity to mitigate attachment-related anxieties, thereby preventing spirals into depression (34). Second, these strengths foster adaptive coping strategies, such as seeking social support or reframing stressors, which disrupt the cycle of isolation and rumination typical of insecure attachment (35). This is supported by our subgroup analysis, where the effect of insecure attachment on depression weakened as inner strengths increased from low to high levels, indicating a dose-dependent protective effect. Interventions targeting inner strengths—such as resilience training or loving-kindness meditation—could serve as cost-effective strategies to mitigate depression risk in working mothers. By integrating such approaches with workplace policies, we can foster environments where working mothers cultivate strengths to buffer attachment-related stressors.

Attachment anxiety is characterized by heightened fears of abandonment, sensitivity to relationship threats, and excessive reassurance-seeking (18). Among working women, these tendencies can interact with stressors such as balancing professional and caregiving roles or managing workplace conflicts, thereby increasing the risk of depression. This aligns with prior research, including Wei et al.'s study (36), showing that attachment anxiety consistently predicts depression, especially in individuals facing multiple intersecting pressures.

In contrast, attachment avoidance, marked by emotional distancing and reluctance to depend on others, did not significantly predict depression in our sample. One possible explanation is that avoidant coping may help working women temporarily compartmentalize stress, effectively masking depressive symptoms in the short term. However, such coping is unlikely to be beneficial in the long run, as avoidance may suppress rather than resolve underlying distress.

These findings highlight the importance of distinguishing between attachment anxiety and avoidance in research on working women's mental health. Interventions should prioritize mechanisms related to attachment anxiety rather than treating insecure attachment as a uniform risk factor.

Furthermore, the study revealed a 38.2% prevalence of depressive symptoms, as measured by the self-reported Outcome Inventory-21 screening tool. This finding is significantly higher than those documented in prior research among Chinese women aged 40–60, where 19.5% reported experiencing depressive symptoms (37). Our finding that 38.2% of working mothers in this study exhibited significant depressive symptoms notably exceeds rates reported in previous studies, such as the 19.5% reported in broader female populations. While methodological and assessment differences may partially account for this discrepancy, it is essential to acknowledge that working mothers constitute a unique subgroup with distinct vulnerabilities. The dual demands of employment and family caregiving can create chronic stress, time pressures, and role conflict, all of which may amplify psychological risks. Recent literature suggests that this intersection of work and family responsibilities intensifies exposure to stressors that are less pronounced in women who do not balance both roles. Therefore, the higher prevalence of depressive symptoms observed in our sample likely reflects, in part, the cumulative impact of these overlapping pressures. This highlights the need for workplace and social support interventions that are specifically tailored to the complex experiences of working mothers.

As noted, the sample was restricted to working mothers residing in urban areas with access to digital resources, while working mothers in rural regions were systematically excluded. This exclusionary sampling strategy introduces constraints on the generalizability of the findings regarding the buffering role of inner strengths in the relationship between insecure attachment and depressive symptoms. Urban working mothers often face distinct contextual stressors—such as higher living costs, faster-paced work environments, and greater access to formal support services (e.g., mental health counseling, childcare facilities)—that may differ substantially from those experienced by rural working mothers, who might contend with limited healthcare access, fewer employment opportunities, and more substantial reliance on informal social networks (38).

In addition, our identification of sociodemographic correlates of depressive symptoms, such as the number of children and educational level, is consistent with previous research. A 2023 study by Garcia et al. on a global sample of mothers showed that having more children was associated with higher levels of stress and depressive symptoms due to increased caregiving responsibilities. Similarly, higher educational attainment has been linked to better stress-coping skills and lower depression risk, as more educated individuals may have access to more resources and better problem-solving abilities (39).

### 4.1 Implications

The findings of this study underscore the crucial role of inner strength as a protective psychological resource in public mental health. It illustrates that among individuals with equally high levels of insecure attachment, those possessing greater inner strengths are less likely to experience depressive symptoms compared to those with lower inner strengths. This highlights the importance of clinical interventions aimed at cultivating inner strengths through training in ten core qualities: truthfulness, perseverance, wisdom, generosity, morality, mindfulness or meditation, patience and endurance, equanimity, determination, and loving-kindness. The significant role of educational levels suggests that individuals with higher education levels tend to exhibit greater inner strengths.

Higher levels of education are generally associated with better access to resources, advanced problem-solving skills, and greater psychological resilience, all of which can contribute to a reduced risk of depression. However, it is also possible that greater educational attainment increases individuals' awareness of psychological distress and mental health issues, potentially leading to more accurate recognition and reporting of depressive symptoms. Thus, while education is typically protective, it may also heighten the ability to identify and acknowledge mental health challenges, which could influence reported prevalence rates. These nuanced effects suggest that education impacts both psychological resources and the recognition of distress, and both factors should be considered when interpreting the relationship between education and depression in working mothers.

### 4.2 Limitations and recommendations for further research

This research bears several limitations that merit explicit acknowledgment. First, the sampling framework was confined to urban and regional demographics in mainland China, specifically urban working mothers with access to digital platforms, thereby excluding rural and lower-income populations without reliable internet access. This limits the generalizability of the findings, as mothers experiencing different socioeconomic or cultural stressors may not be adequately represented. Second, augmenting the sample size and integrating multifaceted moderating variables would enhance the robustness and generalizability of the results. Third, exclusive reliance on self-administered online questionnaires may have introduced biases such as social desirability, recall inaccuracies, and varied interpretation of survey items, especially within culturally sensitive domains like mental health and attachment. While validated instruments and clear instructions were used to mitigate these issues, their influence cannot be entirely excluded. To improve sample representativeness in future research, we recommend broadening inclusion criteria, adopting on-site supplementary approaches, and designing targeted recruitment strategies for populations with limited digital access.

## 5 Conclusions

This study demonstrates that inner strength significantly moderates the relationship between insecure attachment and depressive symptoms among working mothers, highlighting its potential as a modifiable protective factor in public mental health. Theoretical implications highlight the need to integrate attachment and strength-based frameworks in mental health research, as inner strengths are associated with both fewer depressive symptoms and a weaker positive association between insecure attachment and depressive symptoms. This study also found that the prevalence of depressive symptoms among Chinese working mothers is significant, which reminds us that we need to pay great attention to their mental health issues. Clinically, this supports the development of targeted interventions, while socially, policies promoting workplace flexibility and childcare support can foster environments where women cultivate these strengths. Future research should adopt longitudinal designs to track strength development across career-family transitions and explore cultural variations in resilience expression. By prioritizing inner strength cultivation, we can forge inclusive strategies to safeguard working women's mental health.

## Data Availability

The raw data supporting the conclusions of this article will be made available by the authors, without undue reservation.
